# Age-adapted versus age-independent eGFR thresholds to diagnose CKD: integrating the debate and charting a balanced path forward

**DOI:** 10.1093/ckj/sfaf328

**Published:** 2025-10-24

**Authors:** Pierre Delanaye, Priya Vart, Jürgen Floege, Carmine Zoccali

**Affiliations:** Department of Nephrology-Dialysis-Transplantation, University of Liège, CHU Sart Tilman, Liège, Belgium; Department of Nephrology-Dialysis-Apheresis, Hôpital Universitaire Carémeau, Université de Montpellier, Nîmes, France; GIGA Metabolism and Cardiovascular Biology, University of Liège, Liège, Belgium; Department of Clinical Pharmacy and Pharmacology, University Medical Center Groningen, University of Groningen, Groningen, The Netherlands; Division of Nephrology, University Hospital, Rheinisch-Westfälische Technische Hochschule (RWTH) Aachen, Aachen, Germany; Renal Research Institute, New York, NY, USA; Institute of Molecular Biology and Genetics (Biogem), Ariano Irpino, Italy; Associazione Ipertensione Nefrologia Trapianto Renale (IPNET), c/o Nefrologia, Grande Ospedale Metropolitano, Reggio Calabria, Italy

**Keywords:** age-adapted threshold, estimated glomerular filtration rate (eGFR), fixed threshold

## Abstract

The debate over whether to use age-adapted or age-independent estimated glomerular filtration rate (eGFR) cut-offs for diagnosing chronic kidney disease (CKD) reflects a fundamental tension between physiological understanding and clinical pragmatism. Age-adapted proponents highlight robust evidence that GFR declines naturally with age, with many healthy older adults falling below the traditional 60 ml/min/1.73 m^2^ threshold. They argue that fixed cut-offs risk overdiagnosing CKD in the elderly, potentially leading to unnecessary anxiety and treatment, while underrecognizing risk in younger individuals whose GFRs are abnormal for their age. Physiological and longitudinal studies support this view, suggesting age-specific percentiles may better reflect true pathology. Conversely, advocates for age-independent thresholds emphasize that age-related GFR decline is not benign. Large epidemiological studies show that older adults with an eGFR of 45–59 ml/min/1.73 m^2^ are at increased risk of kidney failure, cardiovascular events and mortality, even in the absence of albuminuria. They caution that redefining CKD based on age could exclude high-risk older adults from beneficial therapies and research and that ‘healthy’ reference populations may harbour undiagnosed disease. A balanced approach recognizes the physiological realities of aging while not ignoring the clinical risks associated with reduced kidney function in older adults. Individualized risk prediction tools and shared decision-making can help tailor diagnosis and management. Ultimately, moving beyond arbitrary thresholds toward a patient-centred, risk-based strategy may best serve individuals across their lifespans, ensuring that CKD diagnosis and care are both scientifically sound and clinically meaningful.

## INTRODUCTION

Chronic kidney disease (CKD) is a global health concern, affecting millions of individuals and imposing a significant burden on healthcare systems. The diagnosis of CKD hinges largely on the measurement of glomerular filtration rate (GFR), with the current international consensus—embodied in the Kidney Disease: Improving Global Outcomes (KDIGO) guidelines—defining CKD as an estimated GFR (eGFR) <60 ml/min/1.73 m^2^, irrespective of age, for at least 3 months. However, this seemingly straightforward threshold has become the subject of intense debate. Should the same cut-off apply to a 30-year-old and an 80-year-old, given the well-established physiological decrease in GFR with age? Or does an age-independent definition better serve the goals of early detection, risk stratification and intervention?

This article synthesizes the arguments for, by Pierre Delanaye, and against, by Priya Vart, age-adapted eGFR thresholds, drawing on the latest epidemiological, physiological and clinical trials evidence. Then, Jürgen Floege and Carmine Zoccali offer an expanded, nuanced perspective on how best to reconcile these views in clinical practice.

### The case for age-adapted eGFR cut-offs

#### Epidemiological and physiological rationale

**Figure 1:**
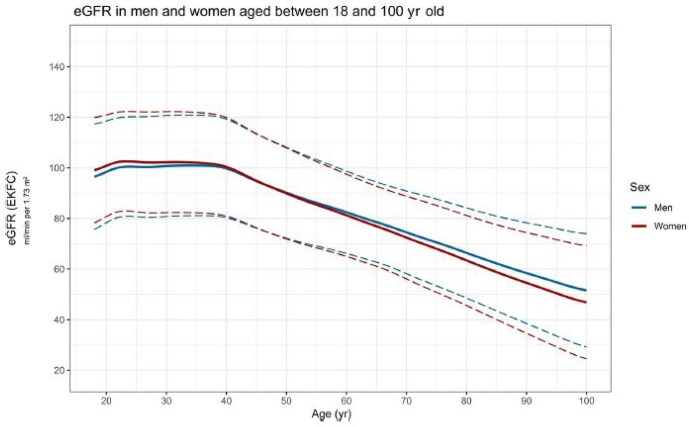
Age-related decrease in GFR among healthy adults [2].

The main argument for age-adapted CKD definitions is rooted in the consistent, robust observation that GFR decreases with age in healthy populations. Cross-sectional studies across continents and populations, using both estimated and directly measured GFR, reveal a strikingly similar pattern: in adults, GFR remains relatively stable until ≈40 years of age, after which it steadily decreases [1, 2]. This phenomenon is not limited to specific ethnicities or methodologies, although minor variations exist; e.g. some Asian populations may exhibit slightly lower normal percentiles [2], i.e. on average, Asian people have values that are lower than the average values seen in other populations, but these values are still considered ‘normal’ for their group. The European Chronic Kidney Disease Burden Consortium, analysing >1.5 million healthy Europeans, reaffirmed this finding, showing that a substantial proportion of healthy individuals >65 years of age have GFR values below the traditional CKD threshold of 60 ml/min/1.73 m^2^ (Fig. 1) [2]. Also, the 90th percentile of eGFR at 65 years of age is 90 ml/min/1.73 m^2^, meaning that only a minority of healthy subjects >65 years of age have a GFR over this value.

Importantly, most of these data are cross-sectional, raising questions about cohort effects and survivor bias. However, the longitudinal Renal Iohexol Clearance Survey (RENIS) in Tromsø, Norway provides compelling evidence that the decrease is not merely an artifact of cross-sectional design. In this study, healthy individuals ages 50–75 years had their GFR measured repeatedly over more than a decade using iohexol plasma clearance, a gold-standard method. The results demonstrated a universal, age-related decrease in GFR, even among those rigorously screened for comorbidities [3]. The 95th percentile of GFR change rate decreased with age, indicating that this is a near-universal phenomenon not restricted to a subset of the population.

Physiological studies further bolster this argument. Landmark work at the Mayo Clinic combined imaging, direct GFR measurement and kidney biopsy in >1300 living kidney donors [4]. They found that while total nephron number and measured GFR decrease with age, the single-nephron GFR remains remarkably stable. This suggests that the age-related decrease in total GFR is not due to pathological hyperfiltration or nephron loss secondary to disease but rather reflects a physiological adaptation—possibly a reduced metabolic demand with aging. Notably, there was no compensatory increase in single-nephron GFR in response to nephrosclerosis, further supporting the notion that this decrease is not inherently pathological.

#### Critique of the fixed threshold

**Figure 2:**
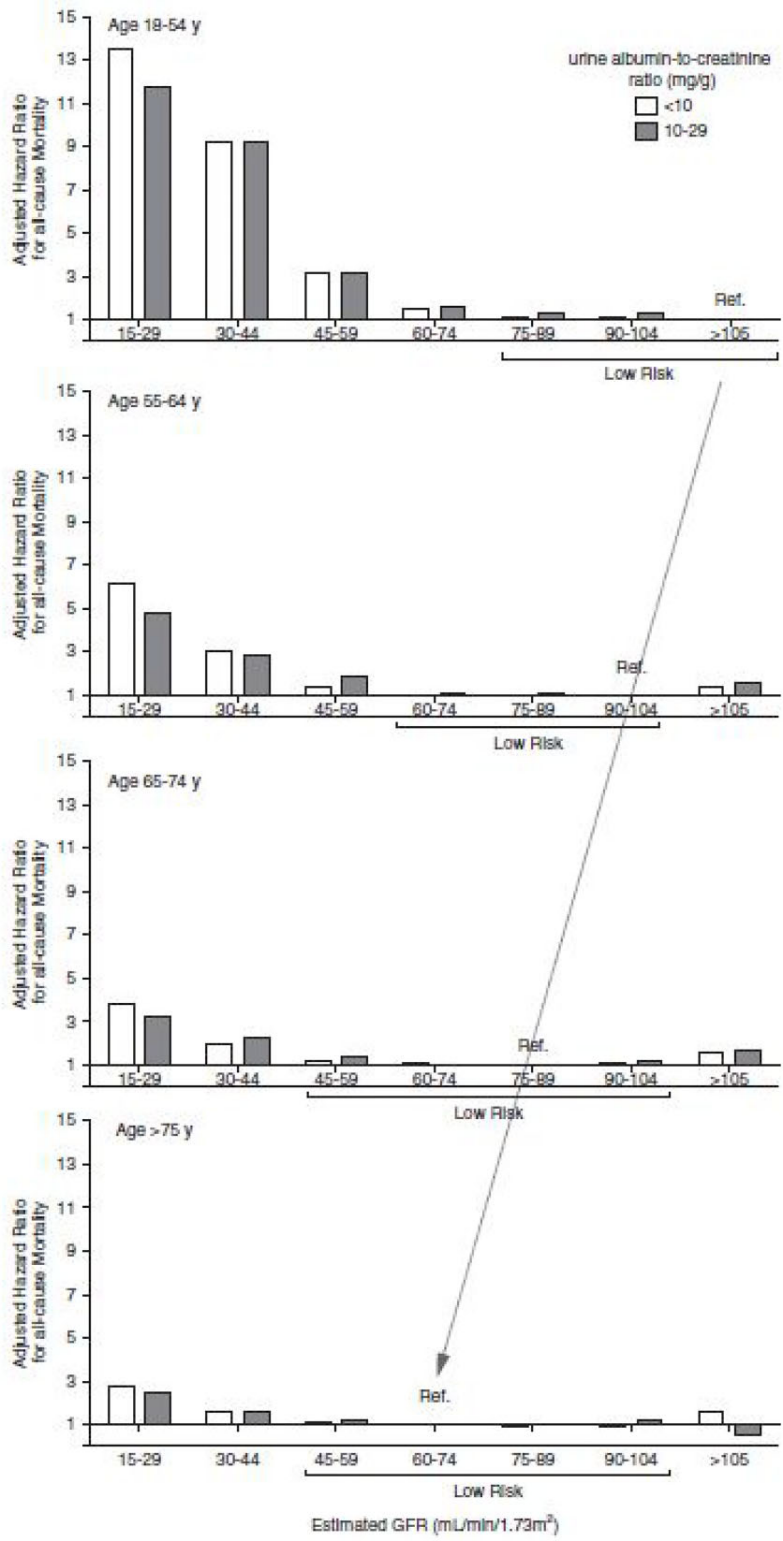
Risk of adverse outcomes by age and eGFR [1].

Proponents of age-adapted thresholds argue that the current fixed cut-off of 60 ml/min/1.73 m^2^, as recommended by the KDIGO [5], risks overdiagnosing CKD in older adults and underrecognizing risk in younger individuals. Studies upon which KDIGO recommendations were based often used a reference group with an eGFR >95 ml/min/1.73 m^2^—a value corresponding to the 95th percentile for healthy individuals >65 years of age. Delanaye argues that when the reference group is adjusted to reflect the lowest-risk GFR range for each age group, the association between an eGFR of 45–59 ml/min/1.73 m^2^ and mortality becomes non-significant in older adults but remains significant in younger adults with an eGFR of 60–74 ml/min/1.73 m^2^ (Fig. 2), an observation supporting the age-adapted threshold approach [1]. This was confirmed in a population-based cohort study in Alberta, Canada, including adults with incident CKD, defined by a sustained reduction in eGFR for >3 months. CKD was defined with either the fixed threshold (<60 ml/min/1.73 m^2^) (*n* = 127 132) or an age-adapted eGFR threshold (<75, 60 and 45 ml/min/1.73 m^2^ for <40, 40–64 and ≥65 years, respectively (*n* = 81 209) [6]. Non-CKD controls (*n* = 90 393) were defined as being ≥65 years of age with a sustained eGFR of 60–89 ml/min/1.73 m^2^ for >3 months and normal or mild albuminuria. Among people with incident CKD with the fixed threshold but not with the age-adapted definition, the majority (*n* = 54 342) were ≥65 years of age and had a GFR of 45–59 ml/min/1.73 m^2^ with normal albuminuria at baseline. In these people, the 5-year absolute risk of kidney failure (≤0.12%) and death was similar in magnitude to the risk observed in the control group. This suggests that the current criteria for CKD that use the same eGFR threshold for all ages may result in overestimation of the CKD burden in an aging population (CKD incidence at age 65–79 years: 2356 versus 714 per 100 000 person-years), overdiagnosis (although the risks of death and kidney failure are similar to controls) and potentially unnecessary interventions in many elderly people who have age-related decreased GFR [6].

The 2024 KDIGO update continues to rely on CKD Prognosis Consortium data, now with >20 million individuals, and uses a reference group with an eGFR of 90–104 ml/min/1.73 m^2^ [7]. While the relative risks for adverse outcomes are smaller in older adults, they remain statistically significant. However, critics argue that these associations are driven by non-GFR determinants, such as the use of cystatin C, which is influenced by factors other than kidney function and is not widely available in clinical practice [8, 9].

#### Potential consequences of age-adapted thresholds

Adopting age-adapted thresholds—or, ideally, GFR percentiles—would have far-reaching consequences. CKD prevalence in older adults would decrease, reducing unnecessary labelling and potential overtreatment. Simultaneously, prevalence would increase in younger populations, where risk may be underestimated under the current definition. For example, an eGFR of 65 ml/min/1.73 m^2^ in a 35-year-old, currently considered normal, would be flagged as abnormal under a percentile-based approach [1].

Critics of this approach sometimes accuse its proponents of nihilism, particularly regarding the care of elderly patients. However, the argument is not to deny care, but to ensure that the diagnosis of CKD reflects true risk and need for intervention. For instance, the EMPA-KIDNEY trial, which demonstrated benefits of sodium–glucose co-transporter 2 (SGLT2) inhibitors in CKD, did not include the population most affected by the overdiagnosis issue—older adults with an eGFR of 45–59 ml/min/1.73 m^2^ and normal albuminuria [10]. Thus the evidence base for treating this group remains limited.

### The case for age-independent eGFR cut-offs

#### Clinical and public health rationale

The discussant in favour of age-independent eGFR thresholds, Priya Vart, argues that the age-related decrease in eGFR is not benign and should not be dismissed as ‘normal aging’. From a patient perspective, the distinction between physiological and pathological decline is irrelevant if the risk of adverse outcomes increases with declining kidney function, especially when interventions exist that may reverse or slow this decline. Large meta-analyses, including an individual-level analysis of 46 cohort studies, have shown that adults ≥65 years of age with an eGFR of 45–59 ml/min/1.73 m^2^ and no to mild albuminuria (CKD stage G3a-A1) are at increased risk of kidney failure, cardiovascular events and death compared with those with a higher eGFR [11]. Even though the relative risk is lower in older adults, the absolute risk remains high due to the prevalence of decreased eGFR in this age group.

Some have argued that the small difference in 5-year risk of kidney failure between older adults with an eGFR of 45–59 ml/min/1.73 m^2^ and those with 60–89 ml/min/1.73 m^2^ does not justify labelling the former as CKD. However, the lifetime risk may be substantially higher, and even the 5-year mortality risk is meaningfully different (8.3% versus 6.1%, a 36% relative increase) [6]. Moreover, older adults with low kidney function have limited reserve, making them more vulnerable to acute kidney injury and other complications [7].

#### Limitations of ‘healthy’ reference populations

A key critique of age-adapted thresholds is the reliance on GFR distributions from ‘apparently healthy’ individuals. The operational definition of ‘healthy’ in these studies is often based on the absence of overt disease but may miss subclinical or undiagnosed conditions, particularly in older adults. Preclinical hypertension and diabetes, as well as lifelong exposures to adverse health factors, can contribute to kidney function decline that is not truly physiological [7]. Thus the observed decrease in eGFR with age may partly reflect undiagnosed pathology, leading to an overestimation of what is ‘normal’ aging.

#### Implications for clinical care and research

Many older adults who would not carry a CKD diagnosis under age-adapted criteria have significant comorbidities such as diabetes and cardiovascular disease. Since CKD independently enhances risk in such patients, both monitoring and treating such patients would have to be adapted [6]. Ignoring these joint effects could be detrimental to their care.

This is particularly important when clinical trial evidence supports the benefits of interventions in such patients. For example, the EMPA-REG OUTCOME trial (mean age 63 years, mean eGFR 74 ml/min/1.73 m^2^) in patients with diabetes and established cardiovascular disease, and the SCORED trial (median age 69 years, median eGFR 45 ml/min/1.73 m^2^) in patients with CKD and diabetes, with or without established cardiovascular disease, demonstrated consistent cardiovascular benefits across levels of eGFR and urine albumin:creatinine ratio. Similarly, intensive blood pressure control has shown efficacy in reducing cardiovascular and renal outcomes even in patients with low or no albuminuria and an eGFR >45 ml/min/1.73 m^2^ [8–10]. Redefining CKD based on age risks excluding older adults from beneficial therapies and research, potentially exacerbating disparities in care.

There are also ethical and practical concerns. Lowering the diagnostic threshold for older adults implies that diminished health is less relevant in this population, potentially reinforcing age-based bias in therapeutic access, resource allocation and research. Older and in particular very old adults are already underrepresented in clinical trials, and age-specific cut-offs could further marginalize this group by reducing the number eligible for study enrolment.

#### Risks of shifting thresholds

The rationale for age-adapted thresholds is to reduce misclassification—decreasing false positives in older adults and false negatives in younger individuals. However, this approach may have unintended consequences. For example, raising the cut-off for younger adults (e.g. to 75 ml/min/1.73 m^2^ for those <40 years of age) could lead to overdiagnosis and overtreatment in a group with low absolute risk while underrecognizing high-risk older adults. In clinical decision-making, absolute risk is often more relevant than relative risk, and older adults tend to have higher absolute risks for adverse outcomes, lowering the number needed to treat to achieve meaningful benefit.

### Toward a balanced, patient-centred approach

The two moderators, Jürgen Floege and Carmine Zoccali, believe that both perspectives offer compelling arguments, and the optimal approach likely lies in integrating their strengths while mitigating their weaknesses.

#### Recognizing physiological aging but not ignoring risk

The physiological decrease in GFR with age is well documented and should inform our understanding of kidney health in older adults. Labelling every older adult with an eGFR <60 ml/min/1.73 m^2^ as having CKD risks overdiagnosis, unnecessary anxiety and potential overtreatment. However, the clinical risks associated with decreased eGFR in this population are real and should not be dismissed. The presence of comorbidities, limited kidney reserve and increased vulnerability to acute insults all argue for continued vigilance and, where appropriate, intervention.

#### Moving beyond arbitrary thresholds

Rather than relying solely on fixed or age-adapted cut-offs, the focus should shift toward individualized risk prediction and management. Tools such as the Kidney Failure Risk Equation, which incorporates multiple variables (age, sex, eGFR, albuminuria, comorbidities), offer a more nuanced assessment of risk and can guide clinical decision-making [12]. This approach allows for the identification of high-risk individuals across the age spectrum, ensuring that those who stand to benefit from intervention are not overlooked.

#### Integrating evidence and clinical judgment

Clinical trial evidence should inform, but not dictate, practice. While trials such as the EMPA-KIDNEY and the DECLARE-TIMI58 have demonstrated benefits of SGLT2 inhibitors in broad populations, their inclusion criteria may not capture all subgroups affected by the overdiagnosis debate. Clinicians must apply evidence judiciously, considering the individual patient’s risk profile, comorbidities and preferences.

#### Ethical and practical considerations

Any shift in diagnostic criteria must be accompanied by efforts to ensure equitable access to care and research participation for older adults. Age should not be a barrier to therapy or study enrolment, and diagnostic criteria should not reinforce age-based bias. At the same time, overdiagnosis and overtreatment in low-risk populations should be avoided.

#### The role of percentiles and reference intervals

The use of age- and sex-specific percentiles for GFR, as advocated by some, offers a promising avenue for refining CKD diagnosis. This approach mirrors practices in other areas of medicine, such as paediatric growth charts and bone mineral density assessment. However, the implementation of such a system requires robust, population-specific reference data and careful consideration of how to integrate percentiles into clinical workflows.

#### Communication and shared decision-making

Finally, clear communication with patients is essential. The diagnosis of CKD carries significant psychological and practical implications, including insurance, employment and quality of life. Clinicians should discuss the meaning of eGFR results in the context of age, comorbidities and overall risk, engaging patients in shared decision-making about monitoring and intervention.

## CONCLUSION

The debate over age-adapted versus age-independent eGFR cut-offs for CKD diagnosis reflects broader tensions in medicine between population-based guidelines and individualized care. The physiological decrease in GFR with age is undeniable, but so too are the clinical risks associated with reduced kidney function in older adults. A balanced approach—grounded in robust risk prediction, individualized management and ethical care—offers the best path forward. As our understanding of kidney disease evolves, so too must our diagnostic criteria, ensuring that they serve the needs of all patients across their lifespans.

## Data Availability

No new data were produced for this article.
